# Insight into the cellular involvement of the two reverse gyrases from the hyperthermophilic archaeon *Sulfolobus solfataricus*

**DOI:** 10.1186/1471-2199-15-18

**Published:** 2014-09-09

**Authors:** Mohea Couturier, Anna H Bizard, Florence Garnier, Marc Nadal

**Affiliations:** 1 Université Versailles St-Quentin, 45 avenue des Etats-Unis, Versailles 78035, France; 2 Institut de Génétique et Microbiologie, UMR 8621 CNRS, Université Paris-Sud, Bât. 409, Orsay Cedex 91405, France; 3 Université d’Evry-Val d’Essonne, Boulevard François Mitterrand, Evry 91025, France; 4 Present address: Department of Molecular Biosciences, The Wenner-Gren Institute, Stockholm University, Stockholm, Sweden; 5 Present address: Institute of Cellular and Molecular Medicine (ICMM), Center for Healthy Ageing (CEHA), University of Copenhagen, Blegdamsvej 3B, København N DK-2200, Denmark; 6 Université Paris Diderot, 5 rue Thomas Mann, Paris 75013, France

**Keywords:** Archaea, Hyperthermophile, Topoisomerase, Supercoiling, Topology, Low temperature, Cytometry, TopR, Quantification

## Abstract

**Background:**

Reverse gyrases are DNA topoisomerases characterized by their unique DNA positive-supercoiling activity. *Sulfolobus solfataricus*, like most Crenarchaeota, contains two genes each encoding a reverse gyrase. We showed previously that the two genes are differently regulated according to temperature and that the corresponding purified recombinant reverse gyrases have different enzymatic characteristics. These observations suggest a specialization of functions of the two reverse gyrases. As no mutants of the TopR genes could be obtained in Sulfolobales, we used immunodetection techniques to study the function(s) of these proteins in *S. solfataricus in vivo*. In particular, we investigated whether one or both reverse gyrases are required for the hyperthermophilic lifestyle.

**Results:**

For the first time the two reverse gyrases of *S. solfataricus* have been discriminated at the protein level and their respective amounts have been determined *in vivo*. Actively dividing *S. solfataricus* cells contain only small amounts of both reverse gyrases, approximately 50 TopR1 and 125 TopR2 molecules per cell at 80°C. *S. solfataricus* cells are resistant at 45°C for several weeks, but there is neither cell division nor replication initiation; these processes are fully restored upon a return to 80°C. TopR1 is not found after three weeks at 45°C whereas the amount of TopR2 remains constant. Enzymatic assays *in vitro* indicate that TopR1 is not active at 45°C but that TopR2 exhibits highly positive DNA supercoiling activity at 45°C.

**Conclusions:**

The two reverse gyrases of *S. solfataricus* are differently regulated, in terms of protein abundance, *in vivo* at 80°C and 45°C. TopR2 is present both at high and low temperatures and is therefore presumably required whether cells are dividing or not. By contrast, TopR1 is present only at high temperature where the cell division occurs, suggesting that TopR1 is required for controlling DNA topology associated with cell division activity and/or life at high temperature. Our findings *in vitro* that TopR1 is able to positively supercoil DNA only at high temperature, and TopR2 is active at both temperatures are consistent with them having different functions within the cells.

## Background

DNA topoisomerases are enzymes responsible for changing the DNA topological state. They are necessarily involved in all DNA processes (transcription, replication, recombination, repair and chromosome segregation) and the resulting appropriate topoisomerase activity modifies the DNA linking number locally and thereby eliminate excess of negative and positive supercoils generated upstream and downstream from the corresponding machinery [1]. Topoisomerases are classified as type I or type II according to whether the transient break in the DNA during their activity is a single-strand (type I) or double-strand (type II) [2,3]. They are further classified as type IA or IB and type IIA or IIB according to the presence of particular motifs in the amino-acid sequence [3].

Reverse gyrase is a particular type IA topoisomerase and it is the only known DNA topoisomerase to introduce positive supercoils into DNA [4-9]. Reverse gyrase was initially discovered in hyperthermophilic and thermophilic *Archaea* and *Bacteria*[10,11]. It was further reported that reverse gyrase was the marker of hyperthermophily and that the corresponding gene(s) may be essential for life at high temperature [12,13]. The positive supercoiling activity of reverse gyrase was proposed to stabilize the DNA duplex against denaturation in extreme temperature environments [9,14], and thereby avoid DNA melting. Positive supercoiling of DNA prevents formation of open complexes [15] as demonstrated by *in vitro* assays of *Sulfolobus* DNA transcription [16]. Homeostatic control of DNA supercoiling involving reverse gyrase has been suggested in hyperthermophilic archaea [17], as it was previously reported for mesophilic bacteria [18,19]. Reverse gyrase acts *in vitro* as a heat-protective DNA chaperone, independently of its supercoiling activity [20]. The helicase-topoisomerase IA chimeric structure of the reverse gyrase [9,21,22] is reminiscent of the physical and functional interaction between the RecQ-like protein and topoisomerase III. This protein pair is found in *Bacteria* and *Eukarya*, and is involved in the DNA repair and recombination needed for genome stability [23-25]. It has therefore been suggested that the reverse gyrase in hyperthermophilic archaea has a role in the maintenance of genome stability [9,26]. Reverse gyrase efficiently anneals complementary single-stranded circles and introduces positive supercoils into DNA containing a bubble and may thus act as a renaturase, contributing to the genome stability by eliminating impaired regions [27,28]. There is indeed diverse evidence that reverse gyrase is involved in recombination and repair. It is specifically recruited to DNA after UV irradiation [29]. The positive supercoiling reaction of reverse gyrase *in vitro* is stimulated by the single-strand DNA binding protein (SSB) [30], a protein that binds to single-strand DNA to prevent its premature annealing during various DNA metabolism processes including replication, recombination and repair [31]; a functional interaction between these two proteins has been demonstrated *in vivo* in the presence of DNA [30]. SSB also enhances the binding and cleavage of UV-irradiated substrates by reverse gyrase, further implicating reverse gyrase in DNA repair [30]. Reverse gyrase inhibits the activity of the translesion DNA polymerase PolY/Dpo4 *in vitro*, possibly thereby preventing the potential high mutational effect of PolY/Dpo4 [32]. Finally, reverse gyrase shows unwinding activity of substrates containing helical junctions, consistent with its involvement in recombination and repair [33].

A gene encoding a reverse gyrase has been also discovered in some moderately thermophilic bacteria: for example in *Nautilia profundicola*, which grows optimally at 45°C [34], the expression of this gene increases substantially at higher temperature. This may confer a selective advantage for such organisms which live close to hydrothermal vents and are therefore subject to frequent and rapid temperature fluctuations [34]. Possibly, reverse gyrase may be important for thermoadaptation rather than the hyperthermophilic lifestyle as such. The situation seems to differ between hyperthermophilic organisms with one and those with two reverse gyrase genes. *Thermococcus kodakaraensis* is a hyperthermophilic organism belonging to the Euryarchaeota *phylum*; it has a single reverse gyrase gene that was shown to be not essential for hyperthermophilic life, except at temperatures above 90°C [35]. By contrast, in the crenarchaeon *Sulfolobus islandicus*, both *topR1* and *topR2* genes were recently demonstrated to be essential [36]. Thus, the two reverse gyrase genes in Sulfolobales, and possibly in all Crenarchaeota containing two, seem to be linked either to the hyperthermophilic lifestyle and/or to other essential functions.

Reverse gyrases clearly have several functions in the cell, probably involving interactions with different partners according to the cellular process. The redundancy of reverse gyrase genes in most members of the Crenarchaeota *phylum* strongly suggests specialization of the two reverse gyrases with TopR1 and TopR2 having different functions. The *topR1* and *topR2* genes in *S. solfataricus* P2 are differently regulated, with different expression patterns according to the growth phase and temperature, and TopR1 is probably involved in the control of the topological state of DNA [17]. Experiments *in vitro* with the two purified recombinant reverse gyrases from *S. solfataricus* showed that they exhibit different enzymatic characteristics and in particular different behaviors with respect to temperature [37].

As both genes are essential in Sulfolobales, we looked for culture conditions revealing differential regulation of the two enzymes to study further their respective roles. We determined the lowest temperature at which only one of the two reverse gyrases exhibits significant enzymatic activity *in vitro.* Then, we tested whether *S. solfataricus* P2 cells at this temperature contain one or both reverse gyrases, to assess whether one or both reverse gyrases are tightly linked to the hyperthermophilic lifestyle. We report that TopR1 is not active at 45°C whereas TopR2 exhibits significant positive supercoiling activity at this temperature. We also report for the first time the number of reverse gyrase molecules per cell: *S. solfataricus* contains approximately 50 molecules of TopR1 and 125 molecules of TopR2 per cell when cells are actively dividing at 80°C. After three weeks at 45°C, there was no cell division nor replication initiation; the abundance of TopR1 was very much lower than at 80°C whereas that of TopR2 is largely unaffected. The cultures were returned to 80°C, and growth ability and replication activity are fully restored and the amounts of TopR1 characteristic of actively dividing cells were recovered. These quantitative findings contribute to elucidating the different roles of reverse gyrase.

## Results and discussion

### TopR2, but not TopR1, remains active at 45°C

**Figure 1:**
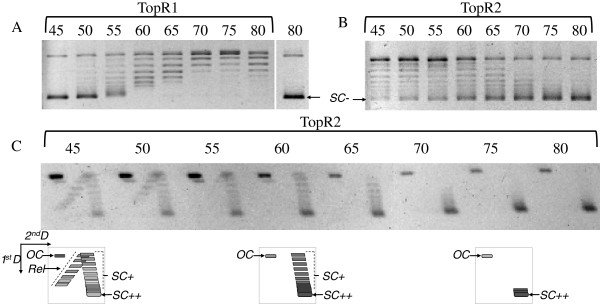
**Effect of low temperature on the activities of TopR1 and TopR2.** Standard positive supercoiling reactions were performed in the presence of a limiting amount of TopR1 **(A)** or TopR2 **(B and C)**. The reaction products were analyzed by one-dimensional gel electrophoresis **(A and B)** and for TopR2 also by two-dimensional gel electrophoresis **(C)**. In panels **A**, **B** and **C**, the incubation temperature is indicated above the gels. Pictures in panel C below the 2D gels schematize the position of the various forms of DNA in 2D gel electrophoresis. SC + and SC ++ indicate the positions of positively supercoiled and highly positively supercoiled DNA, respectively, Rel the position of the relaxed DNA, oc the position of nicked DNA, and SC- the position of negatively supercoiled DNA substrate.

The two reverse gyrases of *S. solfataricus* exhibit different enzymatic properties [37]. Although both enzymes are able to introduce positive supercoils into DNA, TopR2 introduces a higher density of positive supercoils and at a higher rate than does TopR1. This difference is mainly due to the very high processivity of TopR2, whereas TopR1 is distributive. The activity of TopR1 is strictly dependent on the temperature: from relaxation at 60°C the linking number increases progressively with increasing temperature with maximum positive supercoiling being reached at 90°C. By contrast, TopR2 is not active at high temperature *in vitro* (its optimal temperature is around 70°C), but exhibits a significant positive supercoiling activity at 60°C [37]. Thus, the two reverse gyrases can be distinguished according to their activities at different temperatures. Several studies report that various protein machineries in hyperthermophilic organisms of the *Sulfolobus* genus are functional at low temperature: the proton pump [38], the transcription machinery [16], replication and repair DNA polymerases [39,40] and DNA topoisomerase(s) [4]. Consequently, we investigated whether both reverse gyrases of *S. solfataricus* are active at temperatures below 60°C, the lowest temperature previously tested [37]: we performed topoisomerase assays at temperatures from 45°C to 80°C (Figure 1). Enzymes are generally less active at low temperature, so we used a topoisomerase:DNA molecular ratio of 4 to allow weak activities to be detected. The main characteristics of the two enzymes were observed at control temperatures (60°C to 80°C; Figure 1): distributivity for TopR1 and processivity for TopR2 [37]. A significant positive supercoiling activity of TopR1 was observed only at temperatures above 70°C (Figure 1A). TopR1 displayed a weak relaxation activity at 55°C and 50°C but was not active at 45°C (Figure 1A). By contrast, TopR2 was active at 45°C as revealed by the topoisomer profile obtained in one-dimensional gel electrophoresis (Figure 1B). The two-dimensional gel electrophoresis experiments revealed that TopR2 was able to introduce large numbers of positive supercoils into DNA at 45°C (Figure 1C). Although the positive supercoil density at 45°C seemed to be similar to that obtained at 70°C, the population of highly positively supercoiled plasmids was smaller at 45°C (Figure 1C). There was also a wider range of intermediate topoisomers from more or less highly positively supercoiled to negatively supercoiled at lower (particularly at 45°C and 50°C) than higher temperatures (Figure 1C). These observations suggest that TopR2 is less processive at lower temperature. Two DNA polymerases from *S. solfataricus*, PolB1/Dpo1 and PolY/Dpo4 involved in DNA replication and repair, respectively, show also temperature-dependent processivity [39]. In addition, between 45°C and 60°C, we observed that TopR2 produces more nicked DNA than at higher temperatures (65°C-80°C) (Figure 1C). This clearly indicates that low temperature does not inhibit binding or DNA cleavage but does affect the DNA strand passage or religation. Interestingly, overproduction of nicked DNA is not observed for TopR1 (Figure 1A), further evidence of the difference between these two enzymes. In conclusion, in our conditions, TopR2 preserves a significant highly positive supercoiling activity at temperatures down to 45°C whereas TopR1 is not active at such low temperatures. We then tested whether *S. solfataricus* is still alive at this low temperature and studied the reverse gyrase content *in vivo*.

### *S. solfataricus* cells preserve their membrane integrity at 45°C, but cells do not divide

**Figure 2:**
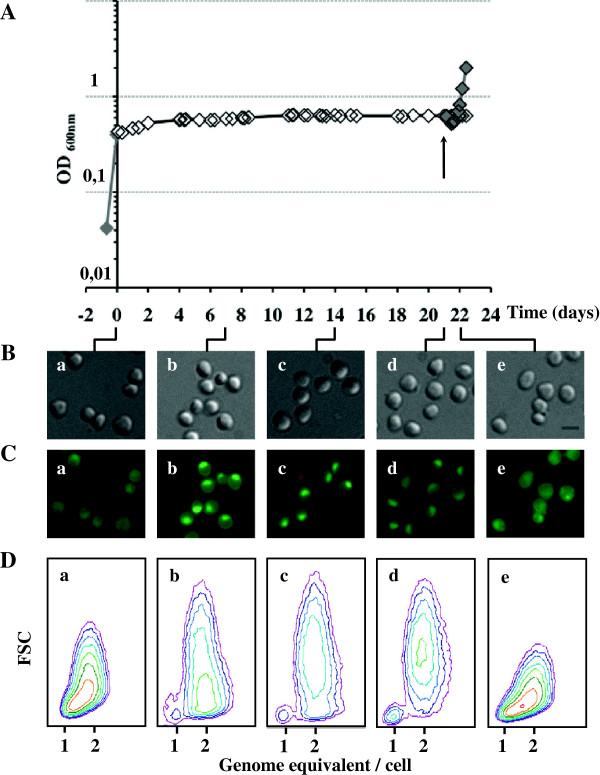
**Cell size, cell integrity and DNA content of *S. solfataricus *transferred from 80°Cto 45°C.** Samples of cells growing exponentially at 80°C (0 h), or after being transferred to 45°C (time points: 7d, 14d and 21d), or transferred back to 80°C (24 h) were collected. The cell density was monitored by measuring the OD _600nm_**(A)** and by flow cytometry **(D)**. The time point (0 h) indicates when 80°C exponentially growing cells (closed gray diamond) were transferred from 80°C to 45°C (open black diamond). The arrow (↑) indicates when cells were transferred back to 80°C (closed gray diamond) after 21 days at 45°C. **(B)** Cell size was measured by phase-contrast microscopy **(a-e)**. Bar, 2 μm. **(C)** Cell membrane integrity was analyzed by fluorescence microscopy. Merged images of Syto 9 (green) and propidium iodide (red) are shown **(a-e)**. The same cells were studied by phase-contrast **(B)** and by fluorescence microscopy **(C)**. DNA content distribution and cell size of *S. solfataricus* cells transferred from 80°C to 45°C for three weeks and back to 80°C **(D)**. DNA content distribution was analyzed as the fluoresence of propidium iodide and the cell size determined by forward light scattering in flow cytometry **(a-e)**.

We first evaluated cell density, cell size, membrane integrity, and cell distribution according to DNA content in cultures at 45°C. We transferred cultures of exponentially growing cells at 80°C to 45°C and maintained the cultures at 45°C for three weeks. Cell density was determined both by measuring optical density at 600 nm (OD_600nm_) and by flow cytometry, and we confirmed that the OD_600nm_ accurately reflects the number of cells as counted by flow cytometry. Despite a small increase of OD_600nm_ during the first two days, the cell density remained roughly constant during the three weeks at 45°C (Figure 2A). The same result was obtained when we changed the culture medium every week (data not shown), showing that this was not due to any modification of the growth medium composition over the long period at 45°C. Thus, the constant OD_600nm_ over a period of three weeks at 45°C was due to the temperature.

Control cells cultured at 80°C and cells transferred to 45°C were examined by phase contrast microscopy: that both presented the same round shape (Figure 2B). Average cell sizes were calculated from measurements of at least 200 cells in each condition. In both conditions, the cells were similarly small: 1.60 μm ± 0.30 for control cells at 80°C and 1.53 μm ± 0.27 for cells transferred to 45°C, independent of the time at which the cells are collected (Figure 2B, panels b-d compared with panel a). This is consistent with a previous report that *Sulfolobus* cell size is not affected by transfer to room temperature or even ice-water [41].

When cells are exposed to an environmental condition different from their optimal growth conditions, cell membrane integrity may be affected. We used the LIVE/DEAD® *Bac*Light™ Bacterial Viability Kit [42], which has been validated for archaea [43] to test membrane integrity. As expected, the exponentially growing cultures at 80°C contained few damaged cells (Figure 2C, panel a); after transfer from 80°C to 45°C and incubation for 21 days, the numbers of cells with preserved membrane integrity were similar to control values (Figure 2C, panels b-d). This indicates that the cells do not lyse at this low temperature, and are resistant to it for a long period.

We used flow cytometry to investigate cell size and cell composition (forward light scattering; FSC) and cell distribution according to DNA content. Cells transferred to 45°C became slightly more heterogeneous, exhibiting a wider range of FSC than control cells (Figure 2D, b, c and d compared with a). There was a symmetric decrease of the FSC range for cells transferred back to 80°C after three weeks at 45°C (Figure 2D, e compared with b, c and d). As microscopy indicated that there was no significant change in the cell size at any time during the experiment, regardless the direction of temperature shift (from 80°C to 45°C or from 45°C to 80°C), the symmetric and reproducible variation of the FSC parameter presumably reflects a change in cell composition. Indeed, the protein concentration declined when cells were transferred to 45°C to half that in 80°C control cells. This probably reflects a change in the balance between degradation, stability and basal synthesis for a particular set of proteins. Changes in the lipid membrane composition is also a plausible explanation because the number of cyclopentane rings in the tetraether lipids of Sulfolobale membranes varies with temperature [44]. The composition and/or the properties of the flexible cell wall may also be modified by the temperature change and affect the FSC.

The 80°C control cells could be separated into three sub-populations according to the DNA content (Figure 2D, panel a) as previously described [45]: a minor population containing one genome equivalent; a significant population with a DNA content between one and two genome equivalents; and the largest cell population containing two genome equivalents. The proportions of the different populations in *S. solfataricus* cultures growing exponentially at 80°C were consistent with the relative lengths of cell cycle phases as previously reported for actively dividing *Sulfolobus* cells [45]. This distribution was drastically modified after a prolonged incubation at 45°C: the cytograms after 7, 14 and 21 days at 45°C evidence the disappearance of cells with a DNA content between one and two genome equivalents; cells containing one genome continue to constitute a small part of the cell population; and the proportion of cells with two genome equivalents increases even further (Figure 2D, panels b-d). The absence of cells with intermediate amounts of DNA reflects the absence of active replication. As the cell density remains constant, it is reasonable to correlate the absence of replication with the absence of cell division at 45°C. The disappearance of cells with intermediate amounts of DNA cannot be explained by degradation of partially replicated DNA because this process would imply either a large increase of the cell population harboring one genome or the lysis of these cells. These two possibilities are clearly not in accordance with our results. Thus, we conclude that the cells having initiated replication, finish their replication, leading cells with two genome equivalents. This completion of replication may be slow, that is consistent with the reduced processivity of the DNA replicases PolB1/Dpo1 and PolY/Dpo4, shown *in vitro* with decreasing temperatures [39,40]. Further evidence for this is that the cell distribution 48 hours after transfer to 45°C is similar to that of control cells except that the population of cells with an intermediate DNA content is slightly but significantly shifted towards a DNA content between 1.5 and 2 equivalent genomes and away from 1 to 1.5 genomes (data not shown). Moreover, the cell density increases slightly during these first two days, consistent with residual cell division. We cannot exclude the possibility that there may have been initiation of replication during the first few days after transfer. It has been shown that during the cell cycle of *Sulfolobus* cultivated at 80°C, a new round of replication can occur only when cell division has been completed [41]. However, we cannot completely exclude the possibility that the absence of cell division was as consequence of there having been no replication activity upon prolonged incubation at 45°C. In any case, the absence of cells with an intermediate DNA content after seven days at 45°C clearly indicates that no new replication started during prolonged incubation at 45°C.

When cells maintained at 45°C for 21 days were transferred back to 80°C, there was a transient small decrease of the OD_600nm_ within a couple of hours (Figure 2A). The OD_600nm_ increased progressively thereafter and the growth curve of the cultures transferred from 45°C to 80°C was similar to that for control cultures at 80°C (Figure 2A). Cell size did not change following the return to 80°C (1.6 μm ± 0.27) (Figure 2B, panel e) and the proportion of damaged cells remained very low, and similar to control values (Figure 2C, panel e). In flow cytometry, the cytogram of cells transferred back to 80°C and collected 24 hours after the up-shift was similar to that of the 80°C control cells (Figure 2D, panel e and panel a, respectively). Indeed, the presence of cells with an intermediate DNA content is fully restored, indicating that normal replication activity was recovered. Hence, the *Sulfolobus* cells maintained at 45°C retained their ability to grow again actively at 80°C even after three weeks at the low temperature. A similar phenomenon has been reported for *Sulfolobus* cells kept for a short time at room temperature and transferred back to their optimal growth temperature [41].

In conclusion, *S. solfataricus* cells are resistant to long periods at 45°C and are able to recover a normal cell activity, *i.e.* cell division and replication, when they are transferred back to 80°C.

### Quantification of TopR1 and TopR2 per *S. solfataricus* cell

**Figure 3:**
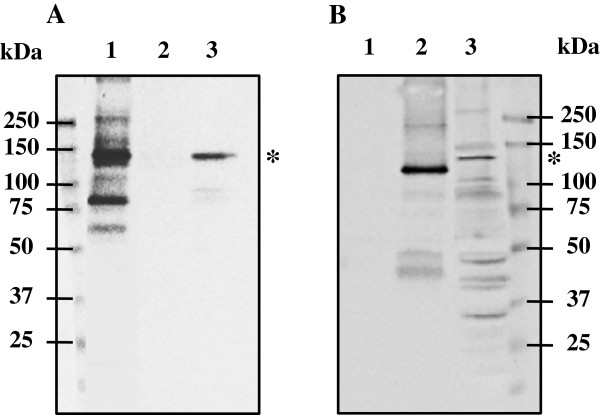
**Specificity of the antibodies raised against the reverse gyrases of *S. solfataricus*.** Western blot analysis of recombinant proteins TopR1 and TopR2 of *S. solfataricus* in lanes 1 and 2, respectively, and of *S. solfataricus* crude extracts in lane 3. Lanes 1-3 were probed with either anti-TopR1 *serum* diluted 1/6000 **(A)** or anti-TopR2 *serum* diluted 1/4000 **(B)**. Molecular weight standards in kDa (Kaleidoscope, BioRad) are indicated near panels **A** and **B**. Lanes 1 in panels A and B contain 0.3 ng and 50 ng of the recombinant TopR1, respectively. Lanes 2 in panels **A** and **B** correspond to 50 ng of the recombinant TopR2. Lane 3 in panel A and lane 3 in panel B contain 10 μg and 50 μg of *S. solfataricus* crude extracts, respectively. Asterisks indicate the signals for TopR1 and TopR2 in crude extracts.

We tested whether the resistance of *S. solfataricus* to relatively low temperatures was associated with changes to the reverse gyrase content. To distinguish between TopR1 and TopR2 in crude extracts, we obtained two specific antibodies (see Materials and methods), each raised against two peptides only found in one of the two reverse gyrases and absent from all other putative proteins of *S. solfataricus*. The pre-immune *sera* recognized some proteins in *S. solfataricus* extracts, but did so only very weakly, and none were similar in molecular mass to the reverse gyrases (data not shown). The specificity of both antibodies was checked with recombinant TopR1 and TopR2, previously purified in our lab [37]. Thus, the anti-TopR1 antibodies recognized purified recombinant TopR1 of *S. solfataricus* (Figure 3A, lane 1) but not the purified recombinant TopR2 (Figure 3A, lane 2); likewise, the anti-TopR2 antibodies recognized the purified recombinant TopR2 of *S. solfataricus* (Figure 3B, lane 2) but not the purified recombinant TopR1 (Figure 3B, lane 1).

**Figure 4:**
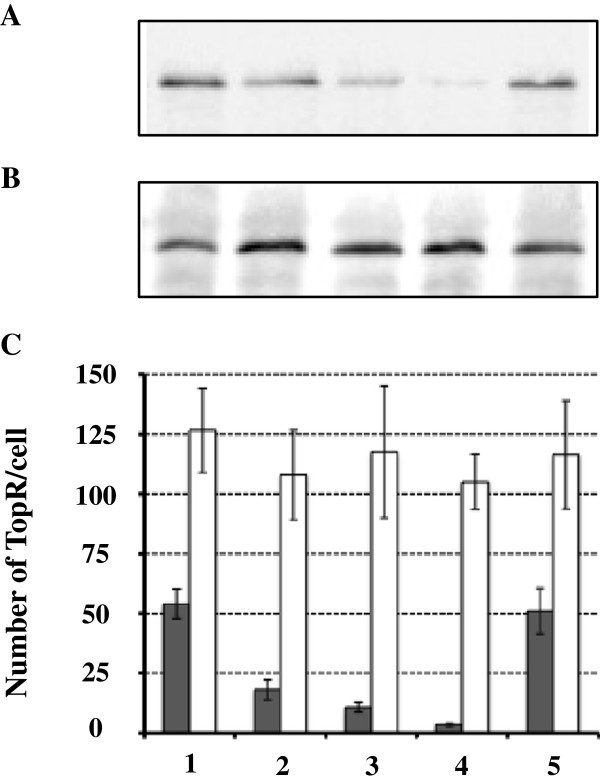
**Reverse gyrase content in *S. solfataricus *cells.** Crude protein extracts were prepared from cells growing exponentially at 80°C (lane 1), transferred to 45°C for 7 days (lane 2), 14 days (lane 3) and 21 days (lane 4) or back to 80°C for 24 hours (lane 5). Aliquots of 10 μg and 50 μg of crude extract were used for detection of TopR1 and TopR2, respectively, by western blotting with anti-TopR1 **(A)** or anti-TopR2 **(B)** antibodies. The specific signals were converted to the number of TopR molecules per cell, shown in panel **C**. The quantification data shown in panel C represent the average of at least three independent experiments.

In crude extracts of *S. solfataricus*, the anti-TopR1 antibodies gave a strong signal for a single band migrating in agreement with the theoretical molecular mass of TopR1 (146.65 kDa) (Figure 3A, lane 3). The strongest signal given by the anti-TopR2 antibodies was with a band migrating in agreement with the theoretical molecular mass of TopR2 (132.6 kDa); it also revealed several additional bands with lower molecular masses, probably corresponding to proteolysis products (Figure 3B, lane 3). The electrophoretic mobility of the protein detected in the *S. solfataricus* crude extract was slightly slower, indicating a higher apparent molecular mass, than that of recombinant TopR2 (Figure 3B, lane 3 versus lane 2). This difference was also observed with antibodies raised against the same specific oligopeptides but produced in another rabbit (data not shown). We conclude that the protein in crude extracts recognized by the antibodies raised against the TopR2 peptides is indeed TopR2 which, like reverse gyrase [46] and numerous *Sulfolobales* proteins [47,48], has post-translational modifications which remain to be elucidated. We used the TopR1- and TopR2-specific *sera* to determine the numbers of the two reverse gyrase molecules present in the different crude *S. solfataricus* cell extracts. We established a calibration curve with known amounts of purified recombinant proteins to allow determination of the absolute number of molecules of each reverse gyrase per cell (Additional file 1: Figure S1). The signal intensity in the various crude extracts was in the same range as that of the calibration, we were able to determine the precise number of TopR1 and TopR2 molecules per *S. solfataricus* cell (Figure 4).

### Number of TopR1 and TopR2 per *S. solfataricus* cell at 80°C

This is the first study reporting the specific detection of two reverse gyrases within the same organism. The immunodetection signal obtained for cells growing exponentially at 80°C (Figure 4A and B, lane 1) corresponded to 54 ± 6 molecules of TopR1 and 127 ± 18 molecules of TopR2 per cell (Figure 4C). These are very low values, but nevertheless in agreement with the very small amounts of the corresponding transcripts reported previously [17].

It has been shown that reverse gyrase has a heat-protective DNA chaperone activity *in vitro*. This activity requires a high protein/DNA mass ratio, of at least 10 [20]. The results we report here indicate a total of approximately 180 molecules of reverse gyrase per cell; if reverse gyrases are regularly distributed along the chromosome, this corresponds to a protein/DNA mass ratio approximately 38-fold lower than required for the *in vitro* chaperone activity. These observations can be reconciled by putative recruitment of reverse gyrase to, for example, sites of damaged DNA as previously discussed [9,29,49]. Similarly, our evidence that there is little reverse gyrase (either TopR1 or TopR2) in each cell is discordant with the requirement for a saturating concentration of the protein for the unwinding activity of TopR1 of four-way DNA junctions [33].

By contrast, our estimations of the amounts of reverse gyrase *in vivo* are consistent with the renaturase activity evidenced in *in vitro*[28]. Indeed the introduction of positive supercoils into DNA containing a bubble of denaturation or the annealing of complementary single strand DNA circles have been evidenced *in vitro* with low protein/DNA mass ratios (0.5 and 0.8, respectively) [28]. These activities are in agreement with thermoprotection of DNA by reverse gyrase: its positive supercoiling activity limits the formation of single-strand regions at high temperature [16]. All *in vitro* experiments have been performed with DNA substrate devoid of any other bound protein; however, *in vivo*, DNA is obviously stabilized by the binding of chromatin proteins, such as Sul7d. The activities of reverse gyrase evidenced *in vitro* may be modulated by DNA binding proteins. Indeed, Sul7d inhibits reverse gyrase activity [50], and the single-strand binding protein, SSB, stimulates both DNA binding and the positive supercoiling of reverse gyrase [30].

### Loss of TopR1 from *S. solfataricus* cells maintained at 45°C

When cells were transferred from 80°C to 45°C for three weeks, the amount of TopR2 per cell remained approximately constant throughout the period at 45°C (Figure 4B and C, conditions 2-4) suggesting either particularly high stability and/or a basal synthesis. The concentration of TopR1 decreased (Figure 4A and C, conditions 2-4): the number of TopR1 per cell declined by two thirds after 7 days, four fifths after 14 days, and to close to the detection threshold after 21 days at 45°C (Figure 4C, conditions 2-4). There was no cell division at 45°C such that the cell number remained unchanged over the three weeks at 45°C, so the loss of TopR1 was necessarily due to a specific but slow degradation. The protein concentration in the crude extracts from cells transferred to 45°C was systematically half that in extracts from 80°C control cells. Consequently, the number of TopR1 molecules per unit protein declined even more than the number per cell, whereas the number of TopR2 molecules per unit protein doubled. Thus, the amounts of the two reverse gyrases were regulated differently in this long-term down-shift experiment.Cells maintained at 45°C for 21 days were able to resume active cell division when shifted back to 80°C. We assayed the two reverse gyrases 24 hours after the shift back to 80°C. The concentration of TopR1 (molecules per cell) returned to baseline control values and that of TopR2 remained unchanged (Figure 4C, condition 5 compared with condition 1). Thus, the ability of the cells to divide correlated with there being appropriate amounts of both TopR1 and TopR2.

The amounts of TopR1 and TopR2 did not change detectably within the first 6 hours following the transfer from 80°C to 45°C (data not shown). The loss of TopR1 observed was slow, and may therefore have been related to the reduced cell activity rather than directly to the temperature change itself. We found that TopR1 is not active at low temperature *in vitro*, consistent with its loss from cultures at low temperature. When the cell activity is reduced, DNA transaction processes are less active, and therefore rigorous control of the topological state of DNA is less critical. At 45°C there was no cell division and no replication, and therefore few or no topological modification of the DNA, so presumably TopR1 is not needed. This implies that TopR1 is the topoisomerase mostly responsible for the DNA topological state in *S. solfataricus*. However, it is also possible that, at low temperature, TopR2 complements the TopR1 deficiency. TopR2 exhibits DNA positive supercoiling activity, which may be sufficient for the residual DNA topological regulation at low temperature. In cells growing at high temperature, TopR1 is presumably required to resolve the frequent modifications of DNA topological state triggered by the cell division activity and enhanced by the high temperature itself.

TopR2 is present both at high temperature (actively dividing cells) and low temperature (non-dividing cells) and it is active at 45°C *in vitro* (Figure 1B and C). The resumption of normal cell activity following the shift back to 80°C after three weeks at 45°C reveals that genome stability was preserved. Although there is little rigorous evidence, reverse gyrase has long been proposed to be involved in maintenance of genome stability [9,26]. As only TopR2 is the only reverse gyrase maintained in cells at 45°C, it may have this function either by stabilizing particular regions of the DNA or by participating in DNA metabolism pathways. After a long period at 45°C, cultures contain few or no cells with between 1 and 2 genome equivalent, although such cells with 1-2 genome equivalent are observed during the 48 h following the transfer to 45°C. TopR2 may contribute to the residual replication activity in cells with 1-2 genome equivalent, such that they progress to containing a whole number (two) of genomes. Transcriptional analysis has shown that the abundance of the *topR2* transcript increases during the G1/S transition phase, suggesting an implication of TopR2 in replication [51]. At 45°C, TopR2 may have short-term functions in both genome stability and replication; subsequently, only the function contributing to genome stability appears to be relevant in the long term as there is no new replication.

TopR1 from *S. solfataricus* inhibits the *in vitro* activity of the translesion DNA PolY/Dpo4, a DNA polymerase involved in response to DNA damage [32]. TopR1 and PolY/Dpo4 interact both *in vivo* and *in vitro*, and presumably this physical interaction mediates the inhibition. However, the interaction was demonstrated with anti-TopR1 antibodies that recognize both TopR1 and TopR2, so it is not clear whether one or both reverse gyrases co-immunoprecipitated with PolY/Dpo4. The topoisomerase domain of TopR1 was reported to be responsible for its affinity for PolY/Dpo4. The topoisomerase domains of *Sulfolobus* TopR1 and TopR2 exhibit a high identity [37], so it is plausible that TopR2 may exhibit significant affinity for PolY/Dpo4. If this were true, it would imply that TopR2, the only reverse gyrase present and active at 45°C, inhibits PolY/Dpo4. At 80°C, there would be competition between the two reverse gyrases, the results of which would depend on their abundance and their respective affinities for PolY/Dpo4.

*Sulfolobus* cells at 45°C have no TopR1 although it is required at 80°C. Presumably, TopR1 is inessential at low temperatures and indispensable at high temperature because the mechanical response of DNA is temperature dependent. *N. profundicola* grows optimally at 45°C and has only one reverse gyrase gene; following exposure to 65°C for two hours, the abundance of the reverse gyrase transcript increases substantially, suggesting that more reverse gyrase is required [34]. This is consistent with the increased frequency of DNA melting at high temperature leading to a greater requirement for reverse gyrase. Possibly, the *Sulfolobus* TopR1 is the functional homologue of the reverse gyrase of *Nautilia*, both proteins exhibiting quantitative variations with temperature. *T. kodakaraensis* also has a single reverse gyrase: a *T. kodakaraensis* mutant deleted for the corresponding gene is viable at temperatures of 60-90°C but not at temperatures higher than 90°C [35]. Possibly, there are compensatory mutations, or other topoisomerases, present in *T. kodakaraensis*, compensating for the absence of reverse gyrase but only at temperatures lower than 90°C. In *S. islandicus*, a strain very closely related to *S. solfataricus*, no mutant viable at 75°C could be obtained for either TopR1 or TopR2 encoding genes, suggesting that both enzymes are essential [36]. In this work, we found that in *S. solfataricus* TopR1 is the major reverse gyrase for the control of the topological state DNA. However, the absence of TopR1 can be compensated at least partially by TopR2, its activity being sufficient to control the minor low topological changes at low temperature. At 75°C, TopR2 cannot complement for the absence of TopR1 because DNA melting is much more extensive, and problematic, at this temperature. As TopR2 may have functions not displayed by TopR1, TopR2 mutants may be lethal. These various observations are concordant in implying that TopR1 is required at high temperature and/or for thermoadaptation.

In this report, we demonstrate that *S. solfataricus* is able to survive at a low temperature (45°C) for a long period, without dividing but with most cells in the culture containing two fully replicated genomes. They are ready to resume a normal cell activity with active cell division as soon as favorable conditions are restored. This property of “cold” resistance may facilitate the spread of *S. solfataricus* to new niches. We also show that the two reverse gyrases are not regulated in the same way indicating that they do have different and possibly overlapping functions in the cells. We provide here evidence, in addition to our previous results [17,37], that TopR1 is important for the regulation of the supercoiling density of the genome, which is affected by replication, transcription and recombination, particularly active in dividing cells at high temperature.

## Conclusions

We report the first quantification of the numbers of reverse gyrase molecules per cell in *S. solfataricus*: in actively growing cultures at 80°C, there are approximately 50 molecules of TopR1 and 125 molecules of TopR2 per cell. At 45°C, *S. solfataricus* does not grow and the reverse gyrase content changes: the amount of TopR1 decreases substantially, although that of TopR2 remains unchanged. These findings *in vivo* are in agreement with the activities of the two enzymes *in vitro*. TopR2 exhibits significant positive supercoiling activity at 45°C, a temperature at which TopR1 is not active. TopR1 is inessential at low temperature but required at high temperature and therefore probably involved in thermoadaptation and/or in DNA transaction processes during active division of *S. solfataricus* cells at 80°C. By contrast, TopR2 is at a constant concentration at both 80°C and 45°C suggesting that TopR2 may be involved in the maintenance of genome stability, particularly in the long term at 45°C when there is no cell division and no replication.

## Methods

### Materials

Tris, glycine, SDS, dimethylsulfoxide (DMSO), acrylamide and bis-acrylamide were purchased from Euromedex. Ponceau S, p-coumaric acid, bromophenol blue, brilliant blue R250, MgSO_4_, Ca(NO_3_)_2_, COSO_4_, CuCl_2_, ZnSO_4_, Na_2_M_0_O_4_, Na_2_B_4_O_7_, FeSO_4_, VOSO_4_, vitamins, HEPES, hydrogen peroxide, Tween 20, luminol, Triton X-100, sucrose, acetic acid, dithiothreitol (DTT), RNase A, and propidium iodide (PI) were obtained from Sigma. KCl, H_2_SO_4_ and KH_2_PO_4_ were from Merck. NaCl was from Fischer scientific. Tryptone peptone and yeast extract were obtained from Difco (Becton Dickinson). Ammonium persulfate (APS), N,N,N’,N’-tetra methyl ethylenediamine (TEMED), bovine *serum* albumin and Bradford reagents were purchased from BioRad. Propan-2-ol, ethanol, sorbitol, HCl, MgCl_2_, MnCl_2_ and (NH_4_)_2_SO_4_ were from Prolabo. Glycerol was obtained from Acros organique.

### Strain

*Sulfolobus solfataricus* strain P2 (DSMZ 1617) was purchased from the Deutsche Sammlung von Mikroorganismen und Zelkulturen in Braunschweig, Germany.

### Reverse gyrase assays

#### Enzymatic assays

The standard reaction mixture contained 50 mM Tris-HCl pH 8.0, 0.5 mM DTT, 0.5 mM EDTA, 20 mM MgCl_2_, 100 mM NaCl, 1.25 mM ATP and 0.15 μg of negatively supercoiled pTZ18R DNA. The purified enzymes [37] were added to the mixture at a molar ratio topoisomerase/DNA of 4, and the mixture was incubated at the indicated temperature for 20 min. The reaction was then stopped by cooling on ice; 0.1% SDS, 25 mg/mL bromophenol blue and sucrose to 15% were added before loading onto the agarose gel.

#### One-dimensional gel electrophoreses

Electrophoresis was performed in 1.2% agarose gels at room temperature in TEP buffer (36 mM Tris, 30 mM NaH_2_PO_4_, 1 mM EDTA, pH 7.8) and run at 3 V/cm for 6 h. Gels were washed in TEP buffer for 15 min, stained with ethidium bromide (2 μg/mL for 30 min) and digitalized under UV light.

#### Two-dimensional gel electrophoreses

TopR2 activity was analyzed after a two-dimensional gel electrophoresis. The first dimension was run at room temperature in a 1.2% agarose gel in TEP buffer at 3 V/cm for 150 min. The gel was then soaked for 30 min in TEP buffer containing 10 μg/mL chloroquin. The second dimension was run in the same buffer, perpendicularly to the first, at 0.9 V/cm for 14 h. The gel was washed in TEP buffer for 30 min and then stained.

### Culture of *Sulfolobus solfataricus* P2 in liquid medium

*Sulfolobus solfataricus* P2 was cultured as previously described [17] at 80°C or 45°C. The cell density was monitored by measuring the optical density at 600 nm (OD_600nm_) and by flow cytometry. Cells cultivated at 80°C and having reached an exponential growth phase (0.3 < OD_600nm_ < 0.6) were transferred to 45°C and maintained at this low temperature for three weeks either in the same medium, or resuspended every six days with fresh medium pre-heated at 45°C. After three weeks at 45°C, the cultures were transferred back to 80°C.

### Phase-contrast and fluorescence microscopy

*S. solfataricus* cells (1.2 × 10^9^ cells) were collected by centrifugation at room temperature at 5000 × *g* for 5 min from cultures at 80°C before the shift to 45°C, at 45°C at various times for three weeks, and again when cells were transferred back to 80°C. The cells were washed with medium then centrifuged at 10000 × *g* for 6 min. Syto 9 and propidium iodide of the LIVE/DEAD *Bac*Light bacterial viability kit (Molecular Probes) were diluted in the culture medium to a final concentration of 7 μM and 10 μM respectively and used to resuspend cell samples to a final concentration of 10^7^ cells/μL. These samples were incubated for 15 minutes at room temperature in the darkness. The cells were observed in a three-dimensional deconvolution microscope (DMIRE2; Leica) equipped with an HCxPL APO 100 × oil CS objective, NA = 1.40 (Leica). The images were captured on a 10-MHz Cool SNAP_HQ 2_ CCD camera (Roper Instruments), with a Z-optical spacing of 0.2 μm. METAMORPH software (Universal Imaging Corp.) was used to acquire Z-series, deconvoluted or not, and treat the images. Only one image with sharp fluorescence from the numerous acquired undeconvoluted Z-series is shown for each condition. In all samples, all the cells incorporated one or both stains and at least 200 cells/sample were examined to determine cell size and cell viability. A minimum of three independent measurements were performed for each condition.

### Flow cytometry

Aliquots of *S. solfataricus* cells were fixed by adding five volumes of 70% ethanol, then diluted with 70% ethanol to obtain a final concentration of 10^8^ cells/mL. The cells were then washed twice with TE buffer (10 mM Tris-HCl pH 7.4 and 10 mM EDTA, pH 8) by centrifugation at 4°C at 10000 x *g* for 6 min. Samples were kept at 4°C during all steps. The cells were then resuspended in TE buffer containing RNase A (10 μg/mL) to a final concentration of 10^8^ cells/mL. The cell preparations were incubated at 37°C for 120 min and centrifuged at 4°C at 10000 × *g* for 6 min. The cells were resuspended in the same volume of TE buffer containing propidium iodide (10 μg/mL) and kept at 4°C overnight in the darkness and analyzed with a PAS III Partec Flow cytometer as previously described [52]. Results are shown as representations of combined forward light scatter (FSC) and DNA content distributions.

### Crude extract preparation

Cell samples (8.25 × 10^9^ cells) were collected as described for microscopy but were cooled immediately on ice, and then centrifuged at 4°C at 5000 × *g* for 5 min. The cells were washed twice with buffer containing 20 mM HEPES and 1 M sorbitol then centrifuged at 4°C at 10000 × *g* for 6 min. The cells were resuspended to a final cell concentration of 4 × 10^10^ cells/mL in extraction buffer (50 mM Tris-HCl pH 7, 15 mM MgCl_2_, 50 mM NaCl, 1 mM DTT, 400 mM sorbitol) and were gently disrupted by the addition of 0.5% Triton X-100 and moderate agitation for 15 min at 4°C. Protein concentrations in the resulting crude extracts were determined by the Bradford method with bovine *serum* albumin as the standard. Aliquots of these protein extracts were denatured with Laemmli buffer and heated at 95°C for 5 min, and resolved by SDS-PAGE.

### Detection of reverse gyrases

Primary antibodies were produced by Eurogentec against two epitopes specific for each of the two reverse gyrases of *S. solfataricus*: PRILYNKQSPTQTEN and EDIQTTMKLLRENIG for anti-TopR1 and GRSKLNIKKYVEDL and YFSEKRKVEEYINNL for anti-TopR2. The choice of these epitopes was based on both the amino acid sequences deduced from the sequences of the genes in *S. solfataricus*[53] and the published structure of reverse gyrase [54]. These peptides are absent from all other CDS of *S. solfataricus*. The peptides were synthesized, linked to hemocyanine and used to immunize rabbits. Proteins were separated by SDS-PAGE (10% acrylamide/0.13% bis-acrylamide), then electroblotted onto nitrocellulose membranes (Whatman Protran BA79) over 1 hour at 4°C in transfer buffer (25 mM Tris-HCl, 192 mM glycine, 15% propan-2-ol). Membranes were stained with Ponceau S, washed with TBS -Tween buffer (20 mM Tris-HCl pH 7.6, 13.7 mM NaCl, 0.1% Tween 20) and blocked with 3% milk in TBS-Tween. Membranes were then probed overnight at 4°C with either 1/6000 anti-TopR1 antibodies or 1/4000 anti-TopR2 antibodies. Membranes were then washed with TBS-Tween and probed for 1 hour at 4°C with 1/20000 horseradish-peroxidase-conjugated anti-rabbit IgG from donkey (GE Healthcare). All dilutions of antibodies were in TBS-Tween. Bound antibodies were visualized with ECL mix (100 mM Tris-HCl pH 8.45, 0.009% H_2_O_2_, 0.225 mM p-coumaric acid, 1.25 mM luminol). The chemiluminescence and Ponceau S signals were captured on a CCD camera (Image Quant LAS 4000, GE Healthcare) and analyzed with the ImageQuant TL (v 7.0) package.

### Estimation of TopR1 and TopR2 number per *S. solfataricus* cell

To determine the number of copies of the two reverse gyrases in *S. solfataricus* cells, we established a relation between ECL signal intensity in the crude extracts and the amount of reverse gyrase. Purified recombinant TopR1 and TopR2 [37] were used as standards. Series of quantities from 0.3 to 50 ng were loaded onto gels with the various crude extracts such that the ECL signal intensities can be compared. The ECL signal intensities obtained for various crude extracts for each condition were compared with the TopR1 and TopR2 calibration curves (Additional file 1: Figure S1) and converted into amounts of reverse gyrase. The amount of protein (in ng) was converted into a number of molecules on the basis of the theoretical molecular weight of the corresponding reverse gyrase. Only membranes for which the Ponceau S staining of the various crude extracts loaded was homogeneous were used for these analyses. Each experiment was performed at least three times independently, and the results reported are the mean values.

## Competing interests

The authors declare that they have no competing interests.

## Authors’ contributions

MC participated in the design of the study, carried out cell culture, microscopy, flow cytometry and western blotting, and helped draft the manuscript. AB carried out reverse gyrase assays. FG participated in the design of the antibodies and establishing the western blot methods, participated in the design of the study and its coordination, and drafted the manuscript. MN participated in the design of the antibodies and establishing the western blot methods, participated in the design of the study and its coordination, and drafted the manuscript. All the authors have read and approved the final manuscript.

## Supplementary Material

Additional file 1: Figure S1 Calibration curves of recombinant proteins TopR1 and TopR2. The purified recombinant TopR1 and TopR2 proteins of *S. solfataricus* were used as standards and the western blots obtained with anti-TopR1 **(A)** or anti-TopR2 **(B)** antibodies are shown. Calibration curves, **C** and **D**, were generated by plotting the ECL signal intensity against the amount of the purified recombinant loaded: from 0.3 to 1.2 ng for TopR1 and from 12.5 to 50 ng for TopR2. The ECL signal intensity obtained for each crude extract was within the range of the ECL signal intensities for three amounts of the purified recombinant TopR, so only these three points are shown. The protein concentrations of the purified fractions of TopR1 and TopR2 were measured by using the Bradford method (BioRad). The purity of the band corresponding either to TopR1 or TopR2 was estimated after SDS-PAGE and gel Coomassie-blue staining. With the specific molecular mass of TopR1 and TopR2 and the % of purity of the corresponding fraction, the precise quantity of the full length proteins, TopR1 or TopR2, was determined as previously reported [37]. Click here for file
