# Supplementary figures and images for: Insight into the cellular involvement of the two reverse gyrases from the hyperthermophilic archaeon Sulfolobus solfataricus
Source: BMC Mol Biol. 2014 Sep 9;15:18. doi: 10.1186/1471-2199-15-18 (PMC4183072; doi:10.1186/1471-2199-15-18)

Fig S1

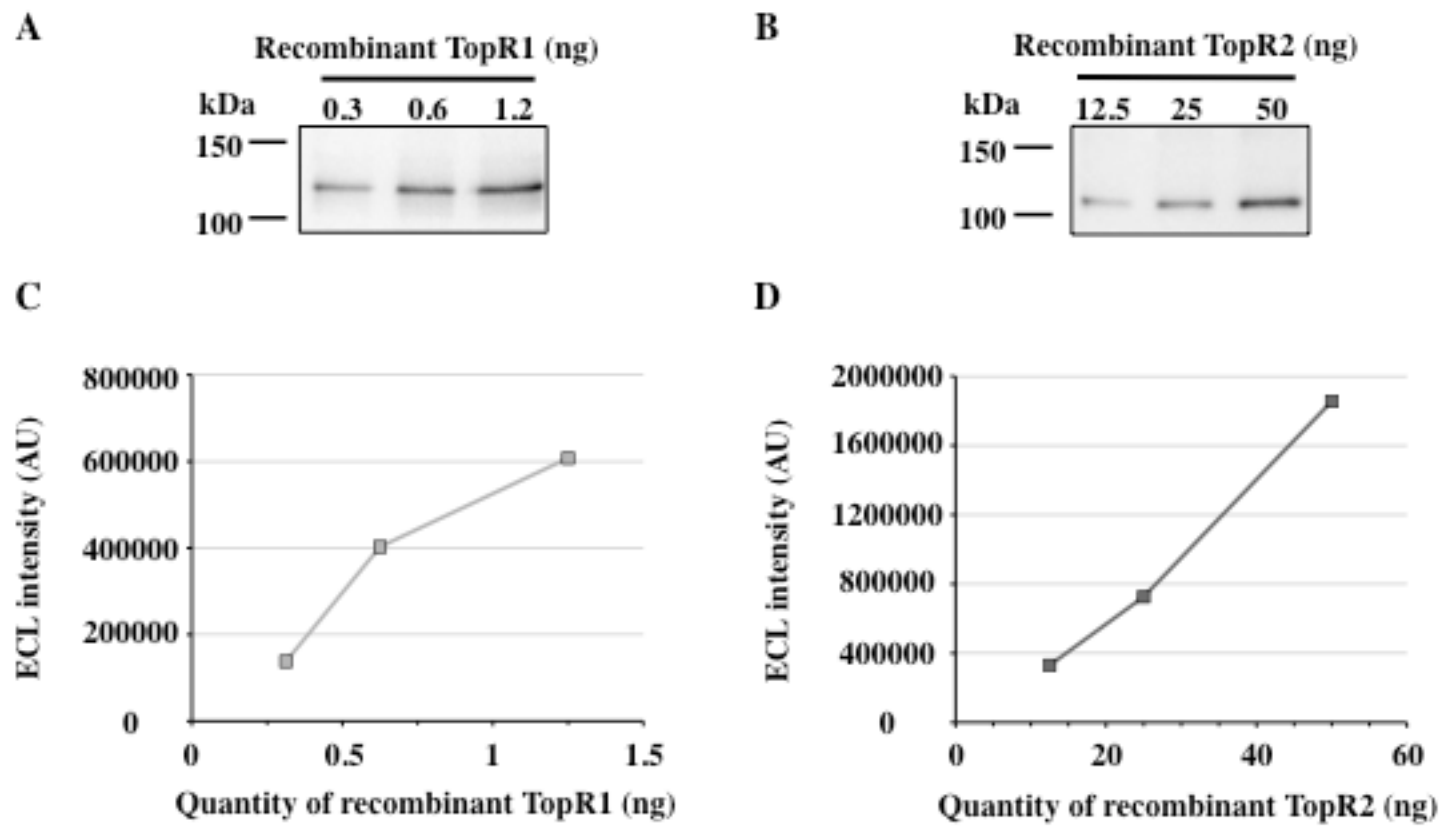

Supplement: Additional file 1: Figure S1 — Calibration curves of recombinant proteins TopR1 and TopR2. The purified recombinant TopR1 and TopR2 proteins of S. solfataricus were used as standards and the western blots obtained with anti-TopR1 (A) or anti-TopR2 (B) antibodies are shown. Calibration curves, C and D, were generated by plotting the ECL signal intensity against the amount of the purified recombinant loaded: from 0.3 to 1.2 ng for TopR1 and from 12.5 to 50 ng for TopR2. The ECL signal intensity obtained for each crude extract was within the range of the ECL signal intensities for three amounts of the purified recombinant TopR, so only these three points are shown. The protein concentrations of the purified fractions of TopR1 and TopR2 were measured by using the Bradford method (BioRad). The purity of the band corresponding either to TopR1 or TopR2 was estimated after SDS-PAGE and gel Coomassie-blue staining. With the specific molecular mass of TopR1 and TopR2 and the % of purity of the corresponding fraction, the precise quantity of the full length proteins, TopR1 or TopR2, was determined as previously reported [37]. [file 1471-2199-15-18-S1.pdf]
